# How a Medicinal Chemistry Project Changed the Way Prostate Cancer is Diagnosed and Treated

**DOI:** 10.1021/acs.jmedchem.5c01593

**Published:** 2025-10-06

**Authors:** Alan P. Kozikowski, Joseph Neale

**Affiliations:** † Prostate Theranostics Inc., 1 Research Court, Suite 450, Rockville, Maryland 20850, United States; ‡ Department of Biology, 8368 Georgetown University, Washington, District of Columbia 20057, United States

## Abstract

Prostate-specific membrane antigen (PSMA), also known as GCPII, has emerged as a significant target in the diagnosis and treatment of prostate cancer. This perspective highlights the journey that led to the development of PSMA-targeted urea-based ligands, culminating in the clinical success of radiopharmaceuticals such as Pylarify and Pluvicto. Originating from research at Georgetown University, early PSMA inhibitors evolved through rational drug design starting from the neurotransmitter NAAG and a phosphonic acid derivative to simplified Glu-urea scaffolds with exceptional inhibitory activity. These ligands enabled the creation of both diagnostic and therapeutic radioligands, as well as emerging small molecule drug conjugates (SMDCs). Ongoing innovation includes bifunctional antigen targeting, alternative PSMA-binding motifs, and efforts to develop brain-penetrant GCPII inhibitors. This mini-perspective reveals how a collaborative chemistry/biology project not only redefined prostate cancer imaging and therapy but also opened new avenues for targeted cancer theranostics.

## Graphical abstract

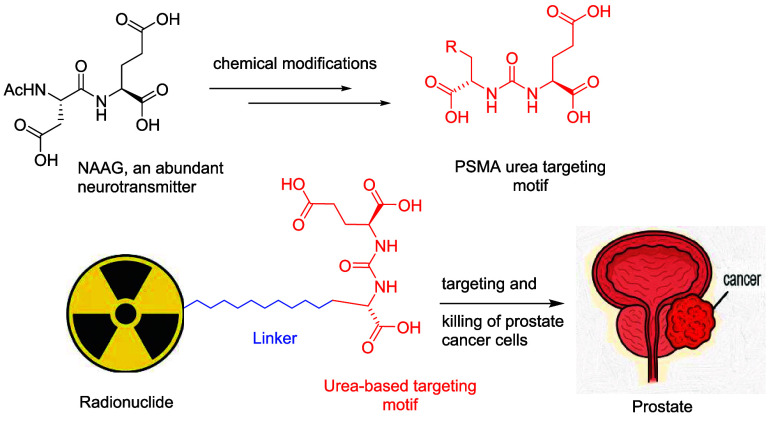

## Significance, Impact, and Innovation

- **Significance**: Discovery of the urea-based PSMA-binding motif transformed prostate cancer care, enabling selective imaging and targeted radioligand therapy.
- **Impact**: Clinical adoption of Pylarify and Pluvicto has redefined the standards of diagnosis and treatment, with widespread patient benefit and a multibillion-dollar market impact.
- **Innovation**: A simple yet versatile scaffold fostered theranostic advances, including radiohybrid ligands, SMDCs, and next-generation inhibitors, inspiring ongoing translational research across the fields of both oncology and neurology.

### What is Prostate Cancer and Patient Numbers

Prostate cancer is recognized as the most common nonskin cancer in men in the US, with almost 233,000 new cases leading to over 29,480 estimated deaths in the U.S. in 2024.^1^ Worldwide, prostate cancer is the second most common cancer in men resulting in over 1.4 million new cases and almost 400,000 deaths in 2022. These statistics have inspired researchers to look for novel therapeutics to treat this devastating disease.^2^

Prostate cancer treatments include a variety of regimens ranging from surgery, radiation therapy, cryotherapy, high-intensity focused ultrasound. androgen (hormone) deprivation, to chemotherapy. Hormone therapy (e.g., abiraterone or darolutamide), chemotherapy (docetaxel), targeted therapy (PARP inhibitors), and immunotherapy using the body’s own immune system (the vaccine called sipuleucel-T) are options for treating metastatic prostate cancers.^3^

Hormonal therapy by androgen ablation and antiandrogen agents remain as the standard treatment for advanced prostate cancer. While hormonal therapy is highly effective at the onset of treatment because prostate cancer growth is normally androgen-dependent, the response to androgen ablation generally persists for less than 18 months, and virtually all patients eventually progress to hormone-refractory prostate cancer (HRPC).^4^ Although recent chemotherapy regimens have demonstrated activity in the treatment of HRPC, none has conferred more than modest improvement in survival.^4^ Advances in our understanding of tumor biology have, however, led to new approaches for the treatment of prostate cancer as well as other cancers, by targeting tumor cells based on their unique protein markers. Several prostate antigens have been identified for both antibody-drug conjugate (ADC) and small molecule drug conjugate (SMDC) targeting, and one of the most studied protein markers is the prostate-specific membrane antigen (PSMA).

### What Are GCPII and PSMA, and How Were They Discovered

W. Gerald Murphy and his collaborators at Memorial Sloan Kettering Cancer Center were the first researchers to identify PSMA in the late 1980s and early 1990s. They generated monoclonal antibodies against human prostate cancer cells, and found that one of these antibodies, known as 7E11-C5.3, was able to bind specifically to a membrane protein, which is highly expressed on prostate cancer cells. Horoszewicz et al. (working with Murphy) published the seminal paper formally describing PSMA as a prostate cell membrane antigen that was expressed at high levels in prostate cancer cells but at much lower levels in normal prostate tissue.^5^ Further molecular cloning and characterization of the PSMA gene (known as FOLH1, for folate hydrolase 1) was completed in the early 1990s by groups including Warren Heston at the Cleveland Clinic, who published a full molecular characterization of this protein.^6^ PSMA has a molecular weight of approximately 84kD and is a type II transmembrane glycoprotein that possesses an intracellular segment, a transmembrane domain, and a large extracellular domain. PSMA is located on the short arm of chromosome 11.^7^

There are other names used for PSMA which reflect its diverse functions in different tissues and organs, for it functions not only as a folate hydrolase but also as a neuropeptidase. Coyle’s group found that PSMA hydrolyzes *N*-acetyl-aspartyl-glutamate (NAAG), a peptide neurotransmitter.^8^ PSMA is a zinc–metallopeptidase member of the M28 family formally designated glutamate carboxypeptidase II (GCPII)^9^ Beyond the prostate, PSMA/GCPII is expressed in kidney, spleen, small intestine, salivary glands, and in the central and peripheral nervous system.^10^

In prostate cancer, PSMA/GCPII works through a number of mechanisms to drive cancer growth. This enzyme cleaves vitamin B9 (folic acid) and other glutamylated substrates to release free glutamate, which in turn activates mGluR receptors on the tumor cells. mGluR activation triggers downstream pro-oncogenic signaling pathways such as PI3K/AKT which promote cell survival while MAPK/ERK activation enhances growth and metastatic potential.^11^ PSMA modulates the tumor microenvironment by altering integrin signaling and facilitating vascular remodeling, thus feeding the tumor with more nutrients and oxygen. PSMA enhances folate metabolism by hydrolyzing polyglutamylated folates to monoglutamylated forms, increasing folate uptake and fueling DNA synthesis, repair, and epigenetic alterations that further drive prostate cancer growth.^12^

GCP II (EC 3.4.17.21) present in the brain cleaves *N*-acetyl-aspartyl-glutamate (NAAG) to *N*-acetyl-aspartate (NAA) and glutamate. The role of GCP II in the brain is to terminate the neurotransmitter activity of NAAG. NAAG is the most abundant dipeptide in the nervous system.^13^ NAAG is released from neurons upon high frequency stimulation as a cotransmitter with glutamate and several different small amine transmitters in a broad spectrum of neuronal pathways. It acts on presynaptic mGluR3 receptors to inhibit further transmitter release. Inhibition of GCPII in the CNS has been effective in animal models of several clinical disorders including inflammatory pain, stroke, TBI, and memory.^14,15^

The initial antibody used to image PSMA, capromab pentetide, is the only prostate cancer imaging antibody approved by the FDA. This antibody has shortcomings in terms of its specificity and sensitivity. An improved antibody that is based upon a humanized version of J591 has been in clinical trials and has shown the ability to image all sites of metastasis, especially bone with nearly 100% specificity and sensitivity.^16^ Other nonprostate sites that express minimal amounts of PSMA are not imaged. The second-generation antibodies have also been used to deliver radionuclides and cytotoxic agents affording therapeutic responses in both preclinical and early clinical trials. One exciting aspect of PSMA is that it is found to be strongly expressed in the neovasculature of all solid tumors, but not normal vasculature.^17^ Therefore, PSMA would appear to be an important target for imaging and therapeutics against solid tumors, and thus work in this area continues at a fast pace. Moreover, the discovery of small molecules able to bind to PSMA with high affinity has offered a game-changing alternative to the use of antibodies.

### The Advent of Small Molecule Inhibitors of PSMA/GCPII

Many of the early studies to design GCP II inhibitors focused on peptide analogs of NAAG.^18^ These efforts by Slusher and Jackson eventually led to the identification of one of the more potent GCPII inhibitors, namely 2-(phosphonomethyl)­pentanedioic acid (2-PMPA). 2-PMPA functions as a competitive inhibitor with a *K*_i_ value of 0.2 nM in a GCPII assay. A structurally related but less polar compound, 2-MPPA, 2-(3-mercaptopropyl)­pentanedioic acid, containing a thiol group in place of the phosphonate, was the first GCPII inhibitor to be tested in a Phase 1 double-blind, placebo-controlled clinical study. No adverse effects were reported (Figure 1).

**Figure 1:**
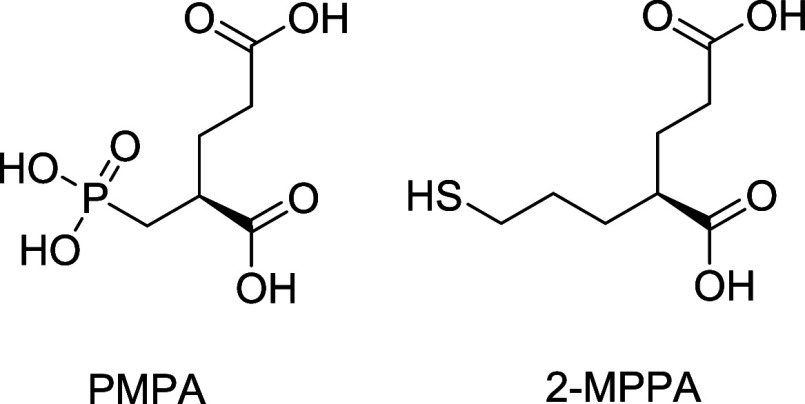
Early GCPII inhibitors PMPA and 2-MPPA.

Our work at Georgetown University on PSMA/NAAG peptidase inhibitors was initiated as a collaboration between Joeseph Neale, in the biology department and Alan Kozikowski, in medicinal chemistry. A sample of PMPA was prepared to explore its activity at mGluR3. In the preparation of this molecule 4,4′-phosphinicobis­(butane-1,3-dicarboxylic acid), abbreviated as PBBDA, was generated as a byproduct.^19^ Biological assays of this compound revealed that it was a potent inhibitor of GCPII (IC_50_ = 21.7 nM) and was a mGluR3 selective agonist with a potency equivalent to NAAG.^19^ In retrospect, one can also arrive at the same structure by carrying out simplification of NAAG as shown in Scheme 1 involving the use of the ketomethylene isostere replacement of the amide, removal of the acetamido group to afford **1**, and symmetrization. While this process leads to the ketone **2** that was found to be poorly active, replacement of the carbonyl group by P­(O)­OH led to the active compound PBBDA. This discovery led us to another design idea, namely, to replace the methylene groups adjacent to the P­(O)­OH group by NH groups, and to restore the carbonyl group, as this would lead to a possible inhibitor comprised of two amino acids linked through the carbonyl group, in other words a urea structure.^20,21^ The result was a simplified, easily synthesized scaffold that did in fact retain potent GCPII inhibitory activity (some compounds in the low nM to pM range) while further SAR defined optimal stereochemistry and revealed the presence of a “hole” outside of the binding pocket, near the left side of the urea as drawn. In fact, we synthesized and screened over 100 of these remarkably simple compounds for their efficacy as GCPII inhibitors in vitro using membrane preparations containing the cloned enzyme and identified a number of compounds that possessed a high level of efficacy. One of them, ZJ43 (Val-urea-Glu) was extensively tested in animal models of inflammatory pain, schizophrenia, traumatic brain injury, Alzheimer’s Disease and memory impairment.^14,15^ It became clear from these early SAR studies that larger substituents could be accommodated in this region of the urea scaffold, thereby enabling the creation of imaging and therapeutic agents decorated with appropriate radionuclides or nonradioactive chemical warheads.^22^

**Scheme 1:**
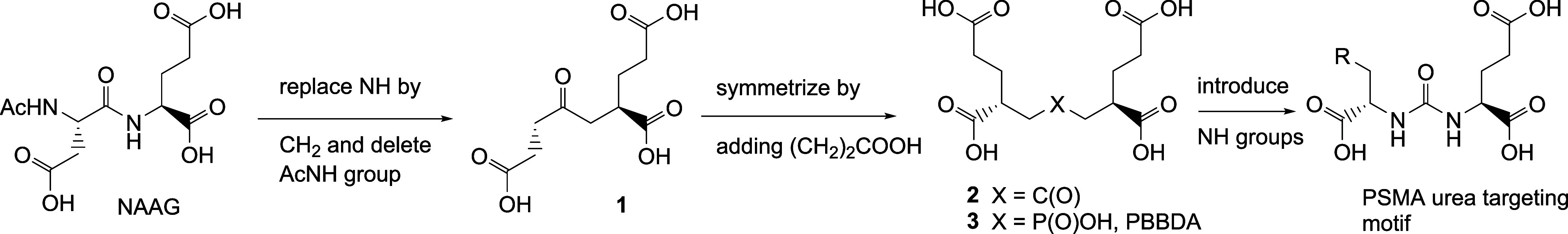
From NAAG to the PSMA Urea Targeting Motif

Employing this rationale, with the notion of using these compounds for prostate cancer imaging, Kozikowski designed and synthesized a cysteine-glutamate urea scaffold, which could be alkylated at the cysteine thiol group to introduce a fluorinated benzyl group. This led to the compound DCFBC [*N*-[*N*-((*S*)-1,3-dicarboxypropyl)­carbamoyl]-4-[^18^F]­fluorobenzyl-l-cysteine], an early precursor to Pylarify (Figure 2).^23^ The nonradioactive version of this compound along with precursor molecules were prepared at Georgetown University, then shipped to Johns Hopkins University, where it was radiolabeled and tested. This work resulted in several publications with Dr. Martin Pomper, an expert in PET, that demonstrated robust imaging in animal models and human subjects.^22–29^

**Figure 2:**
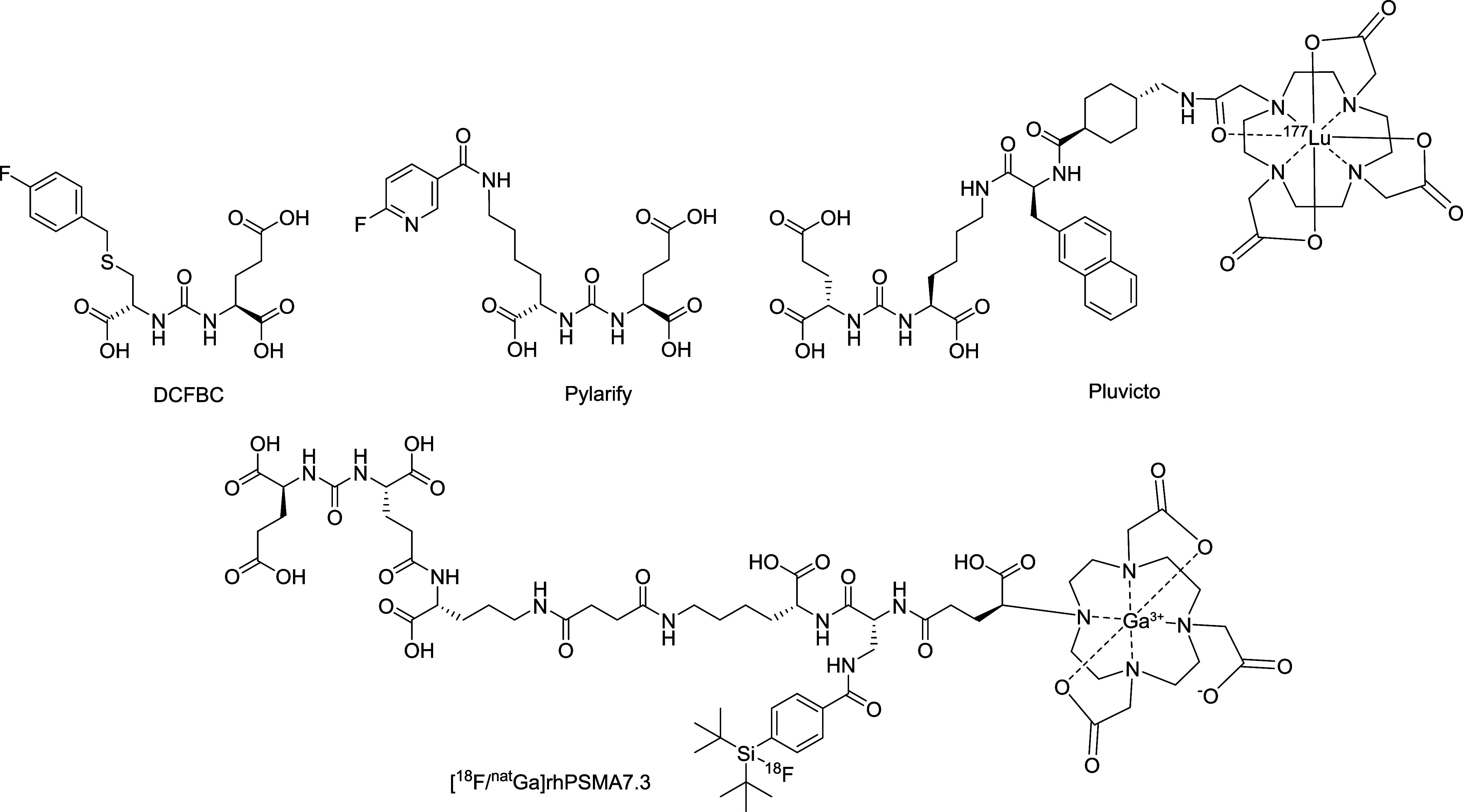
Structures of DCFBC, Pylarify, Pluvicto and rhPSMA7.3.

Further exploiting the “hole” concept, Kozikowski’s design included lysine-glutamate (Lys-Glu) urea motifs with terminal amines suitable for conjugation to radiolabeled moieties.^30^ This scaffold ultimately formed the basis for Pylarify, the top-selling diagnostic agent for PSMA-positive prostate cancer, which in 2024 generated over $1 billion in sales for Pylarify’s manufacturer Lantheus.^31^ A similar urea design was also adopted by Novartis in the development of Pluvicto, a lutetium-177-labeled therapeutic agent for treatment of prostate cancer, now generating over $1.4 billion in annual sales.^32^ Of further note is the development of radiohybrid PSMA (e.g., rhPSMA7.3) ligands. These are dual-labeled agents that combine a silicon fluoride acceptor for ^18^F PET imaging with a DOTA chelator for incorporation of therapeutic radionuclides like ^177^Lu or ^225^Ac, thus enabling both diagnosis and treatment with the same molecular scaffold.^33^ This theranostic design offers high PSMA affinity, excellent imaging resolution, and personalized treatment potential while simplifying clinical logistics.

### What Are the Limitations of Radioligand Therapy and How Do We Do Better

Despite the remarkable efficacy of PSMA-targeted radiopharmaceutical therapy (RPT) in imaging and PSA (prostate-specific antigen) response, the current clinical data are showing certain limitations, including cancer recurrence and progression, and radiation-induced toxicity. Moreover, the need for special equipment and facilities to perform the imaging and therapeutic work is also a limiting factor. The disposal of radioactive waste is yet another cost that must be considered in employing such treatments. As a result, scientists are now focusing on the development of small molecule drug conjugates (SMDCs).

Of considerable current interest is the development of SMDCs in the treatment of cancers. In contrast to antibody drug conjugates, or ADCs, the SMDCs are more amenable to the standard methods of organic synthesis, generally smaller in size, and less likely to be immunogenic. SMDCs may also clear from circulation more rapidly, reducing systemic exposure and off-target toxicity. The linker itself can be tuned to tumor specific enzymes. The smaller size of the SMDCs also provides the advantage of providing more ready entry into the cancer cells, thus permitting their use against solid tumors. Micrometastases and poorly vascularized regions represent a major challenge for ADCs. For example, a trifunctional molecule comprised of monomethyl auristatin E as the chemical payload, with the Lys-Glu PSMA targeting group, and the transthyretin (TTR) ligand AG10, resulted in a conjugate with lower toxicity, an improved half-life, and a better therapeutic efficacy in comparison to docetaxel when tested in a xenograft mouse model of prostate cancer (Figure 3).^34^ It is conjectured that the binding of the TTR ligand to TTR in blood increases the effective molecular weight of these molecules, thereby improving the half-life and penetration into the tumor tissue.

**Figure 3:**
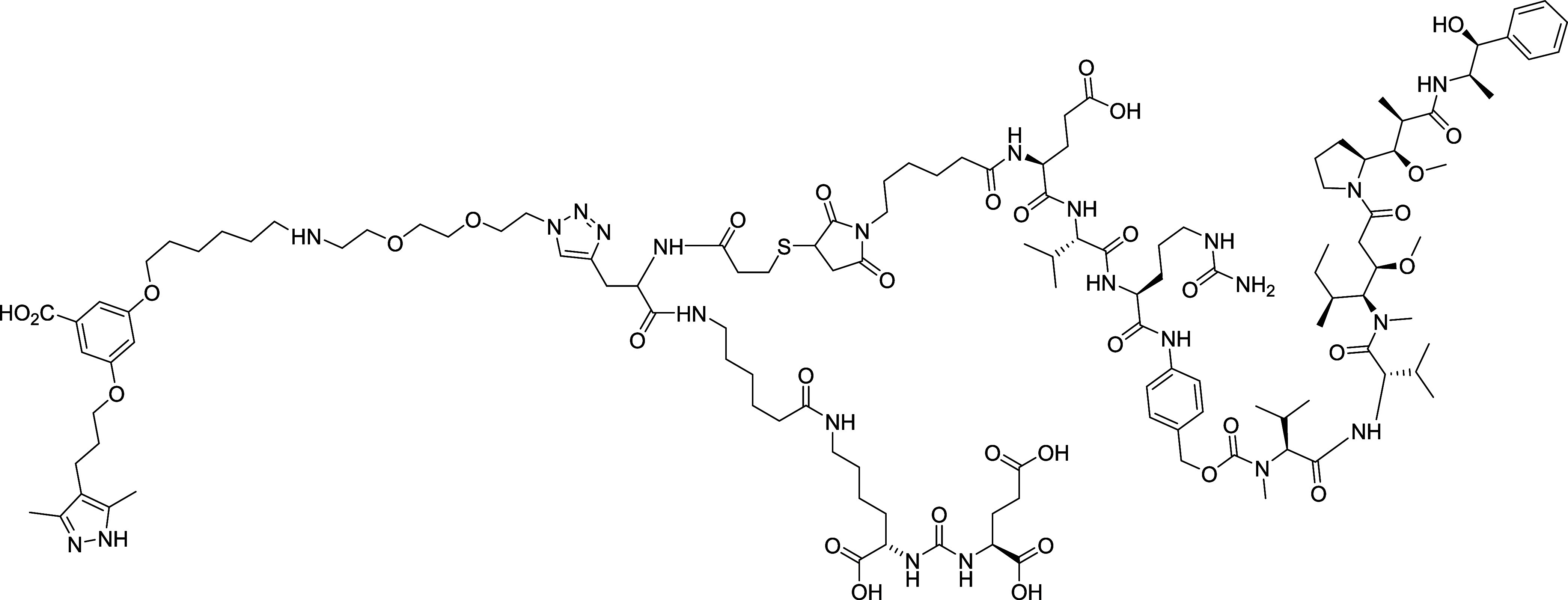
Small molecule drug conjugate EVCit-TFM containing the cleavable glutamic acid–valine–citrulline (EVCit) linker, the hydrophilic transthyretin (TTR) binding ligand, AG10, and monomethyl auristatin E as the chemical payload forming a trifunctional molecule (TFM) of improved therapeutic efficacy.

Considerable ingenuity has also gone into the nature of “cleavable linkers” as in most cases the chemical warhead must be released to reach its ultimate target after the SMDC undergoes endocytosis. While there are a number of unique SMDCs that have been created to date, none have been advanced to human clinical studies. In contrast there are over 17 approved ADCs on the global market, such as Kadcyla (Trastuzumab Emtansine, T-DM1), developed by Roche. Kadcyla is a HER2-targeted antibody-drug conjugate (ADC) which contains the humanized anti-HER2 IgG1, trastuzumab, covalently linked to the microtubule inhibitory drug, a maytansine analog, via a stable thioether linker. This ADC was approved by the FDA in 2013 to treat metastatic breast cancer.^35^ It is of interest to note that maytansine was never approved for stand-alone clinical use due to severe systemic toxicity and its poor therapeutic index. Importantly, the toxicity profile of PSMA-SMDCs should be more favorable than that of ADCs, based on a shorter residence time and/or more rapid and uniform diffusion into the tumor mass compared with normal organs such as kidneys, lacrimal glands, and salivary glands.

### Can We Avoid Off-Target Issues like Xerostomia

Pluvicto causes xerostomia due to off-target radiation in salivary glands. Attempts to reduce uptake of the radioligand in the parotid glands using external cooling has been found ineffective.^36^ In another effort to circumvent this problem, tris­(1-propoxycarbonylethyl) 2-phosphonomethylpentanedioate (TrisPOC, JHU-2545), a prodrug of 2-PMPA, was synthesized to enhance 2-PMPA delivery to nonmalignant tissues. When tested in rodents, JHU-2545 resulted in approximately 3- and 53-fold greater exposure of 2-PMPA in rodent salivary glands and kidneys versus prostate tumor xenograft. Furthermore, JHU-2545 was found to block rodent kidney and salivary gland uptake of the PSMA PET tracers [^68^Ga]­Ga-PSMA-11 and [^18^F]­F-DCFPyL by up to 85% with little effect on the tumor.^37^ While these results are encouraging, this research needs to be extended to human clinical trials to test its value.

A further possibility for exploration is to create prostate cancer targeting agents that show selectivity for antigens that are expressed by prostate cancer cells but not by salivary glands. One such antigen is STEAP1 that is overexpressed in >90% of prostate cancers, particularly metastatic and treatment-resistant tumors. STEAP1 is localized to the cell surface, thus making it accessible to antibodies or SMDCs, while showing little expression in normal tissues and minimal expression in salivary glands, perhaps offering a better target than PSMA.^38^ The creation of bifunctional molecules capable of binding to multiple antigens can also be considered.

### Where Are We Going Next? Can We Do Better Than the Urea Targeting Motif

While the urea motif has proven quite flexible in creating a host of PSMA targeting radioligands, we consider strategies for improvement. Does the Glu-Urea-Lys group provide the very best PSMA targeting group, or can we find alternatives? As we have shown previously, the Lys residue can be replaced by aminoadipic acid to obtain compounds of low nM inhibitory potency (Figure 4).^39^ In fact, in our recent, unpublished work, we have observed that using this particular amino acid, coupled through a linker to a known PARP inhibitor, we were able to achieve picomolar inhibitory activity against PSMA. However, to determine if one amino acid-urea-amino acid motif is better than another, additional screening assays are needed, including appropriate ADME/PK studies. The next generation of chemists are encouraged to think out of the box and are challenged to identify better PSMA targeting motifs.

**Figure 4:**
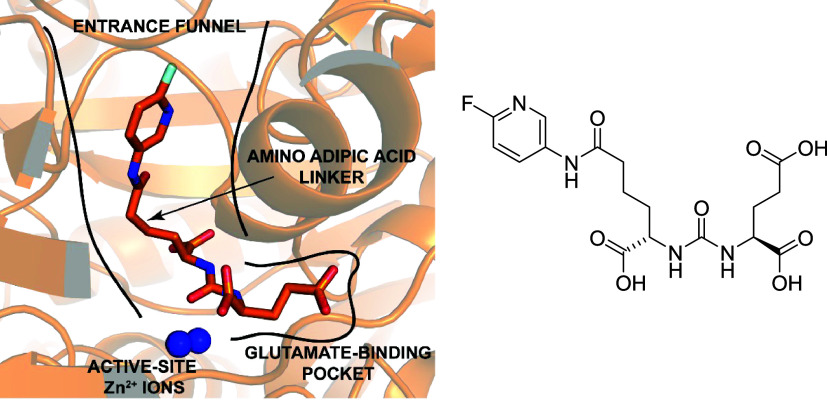
X-ray structure of an aminoadipic acid-urea-glutamate ligand in complex with PSMA (left panel). This structure was rendered using PyMOL 3.1 and PDB file 6HKJ. The structure of the urea ligand is shown on the right.

As noted earlier, compounds like the urea based ZJ43 were effective in animal models of clinical disorders, they failed to penetrate the blood-brain-barrier with sufficient efficacy as to be useful drugs for clinical trials. The urea motif is simply too polar and its prodrug/ester forms provided no solution. Again, this presents an interesting opportunity in drug design, for the invention of brain penetrable GCPII inhibitors could potentially apply to the treatment of autism spectrum disorders, schizophrenia, cognitive impairment, neuropathic pain, epilepsy, and traumatic brain injury.

Opportunities abound for the next generation of chemists and biologists to create a new generation of PSMA/GCPII inhibitors, and significant potential rewards are there for both patients and researchers. This mini-perspective is meant to serve as a call to arms for other chemists to join in the design and discovery of important theranostics to treat prostate cancer.

## Abbreviations Used

ADC: antibody drug conjugate
DCFBC: *N*-[*N*-((*S*)-1,3-dicarboxypropyl)­carbamoyl]-4-[^18^F]­fluorobenzyl-l-cysteine
DCFPyL: 2-(3-{1-carboxy-5-[(6-fluoro-pyridine-3-carbonyl) amino]­pentyl}­ureido)­pentanedioic acid
EVCit-TFM: glutamic acid–valine–citrulline trifunctional molecule
FOLH1: folate hydrolase 1
GCPII: glutamate carboxypeptidase II
HER2 IgG1: human epidermal growth factor receptor immunoglobulin G1
2HRPC: hormone-resistant prostate cancer
MAPK/ERK: mitogen-activated protein kinase/extracellular signal-regulated kinase
MPPA: 2-(3-mercaptopropyl)­pentanedioic acid
NAAG: *N*-acetylaspartylglutamate
PARP: poly­(ADP-ribose) polymerase
PBBDA: 4,4′-phosphinicobis­(butane-1,3-dicarboxylic acid)
PET: positron emission tomography
PI3K/AKT: phosphoinositide-3-kinase/AKR mouse stock thyoma
2-PMPA: 2-(phosphonomethyl)­pentanedioic acid
PSMA: prostate specific membrane antigen
rhPSMA: radiohybrid PSMA
RPT: radiopharmaceutical therapy
SMDC: small molecule drug conjugate
STEAP1: six-transmembrane epithelial antigen of the prostate 1
TBI: traumatic brain injury
TrisPOC: tris­(1-propoxycarbonylethyl) 2-phosphonomethyl-pentanedioate
TTR: transthyretin
